# Left Hemisphere Dominance for Negative Facial Expressions: The Influence of Task

**DOI:** 10.3389/fnhum.2021.742018

**Published:** 2021-09-17

**Authors:** E. Darcy Burgund

**Affiliations:** Department of Psychology, Macalester College, Saint Paul, MN, United States

**Keywords:** hemisphere, facial expression, emotion, faces, negative, divided-visual field

## Abstract

Major theories of hemisphere asymmetries in facial expression processing predict right hemisphere dominance for negative facial expressions of disgust, fear, and sadness, however, some studies observe left hemisphere dominance for one or more of these expressions. Research suggests that tasks requiring the identification of six basic emotional facial expressions (angry, disgusted, fearful, happy, sad, and surprised) are more likely to produce left hemisphere involvement than tasks that do not require expression identification. The present research investigated this possibility in two experiments that presented six basic emotional facial expressions to the right or left hemisphere using a divided-visual field paradigm. In Experiment 1, participants identified emotional expressions by pushing a key corresponding to one of six labels. In Experiment 2, participants detected emotional expressions by pushing a key corresponding to whether an expression was emotional or not. In line with predictions, fearful facial expressions exhibited a left hemisphere advantage during the identification task but not during the detection task. In contrast to predictions, sad expressions exhibited a left hemisphere advantage during both identification and detection tasks. In addition, happy facial expressions exhibited a left hemisphere advantage during the detection task but not during the identification task. Only angry facial expressions exhibited a right hemisphere advantage, and this was only observed when data from both experiments were combined. Together, results highlight the influence of task demands on hemisphere asymmetries in facial expression processing and suggest a greater role for the left hemisphere in negative expressions than predicted by previous theories.

## Introduction

Facial expression recognition is an important ability that provides crucial information about others’ internal states. The extent to which emotional facial expressions are differentially processed by areas within the right and left hemispheres of the brain is a topic of significant debate. Much of this debate has focused on three theories of hemisphere asymmetries in emotion processing. The Right Hemisphere Hypothesis (RHH; e.g., Borod et al., 1998) argues that the right hemisphere is dominant for the processing of all emotional stimuli and is therefore dominant for the processing of all emotional facial expressions. The Valence Specific Hypothesis (VSH; e.g., Adolphs et al., 2001), in contrast, proposes that the right hemisphere is dominant for the processing of negative emotions but the left hemisphere is dominant for the processing of positive emotions. According to this theory, negative facial expressions such as anger, disgust, fear, and sadness produce right hemisphere dominance, while positive facial expressions such as happiness and surprise produce left hemisphere dominance. A similar theory, the Approach/Withdraw Hypothesis (AWH; e.g., Harmon-Jones, 2004), argues that the right hemisphere is dominant for withdrawal emotions and the left hemisphere is dominant for approach emotions. The difference between this theory and the VSH is that it classifies anger as an approach (left hemisphere) emotion rather than a negative (right hemisphere) emotion.

Previous research on facial expression processing is mixed in its support of these theories. Some studies observe results that align with the RHH (Bourne, 2010; Ross et al., 2013; Innes et al., 2016; Damaskinou and Watling, 2018; Kajal et al., 2020); other studies observe results that are more in line with the VSH (Jansari et al., 2011; Kumar and Srinivasan, 2011; Prete et al., 2014a; Lichtenstein-Vidne et al., 2017); few studies, if any, provide direct support for the AWH. As such, a growing number of researchers have suggested that the truth may be some combination of the RHH and VSH perspectives (Killgore and Yurgelun-Todd, 2007; Rahman and Anchassi, 2012; Abbott et al., 2013; Najt et al., 2013; Prete et al., 2015a, b, 2018; Lai et al., 2020).

Critically, despite the important differences between the theories, the RHH, VSH, and AWH are similar in that they all predict right hemisphere dominance for the negative facial expressions of sadness, fear, and disgust. This effect is observed by many studies (e.g., Bourne, 2010; Kumar and Srinivasan, 2011; Rahman and Anchassi, 2012; Innes et al., 2016; Damaskinou and Watling, 2018; Kajal et al., 2020; Hausmann et al., 2021). Nonetheless, some studies have observed left hemisphere dominance for one or more of these expressions under particular experimental conditions (Prete et al., 2014b, 2015a; Burt and Hausmann, 2019; Bublatzky et al., 2020; Lai et al., 2020; Stanković and Nešić, 2020). As such, regions within the left hemisphere may play a greater role in processing facial expressions than predicted by the RHH, VSH, or AWH theories.

One factor that appears to play a role is the number of facial expressions included in the task. A study by Abbott et al. (2014) suggests that left hemisphere regions are more involved when the task requires recognizing a larger compared to a smaller number of expressions. Patients with right or left hemisphere damage and control subjects performed three blocks of an identification task in which they labeled emotional facial expressions. The number of expressions to be identified varied from two (happy and sad) in the first block, four (happy, sad, surprised, and disgusted) in the second block, and all six basic emotions (happy, sad, surprised, disgusted, angry, and fearful) in the third block. When two facial expressions were tested, patients with right hemisphere damage were impaired compared to patients with left hemisphere damage for both happy (positive) and sad (negative) expressions, in line with the RHH. When four or six facial expressions were tested, however, right and left hemisphere damage patients were equally impaired compared to controls for positive and negative emotions. Thus, areas within the left hemisphere contribute to facial expression processing to a greater extent when more facial expressions are included in the task, and therefore, number of expressions included could play a role in producing left hemisphere dominance in negative facial expression processing. Importantly, however, more of the studies observing left hemisphere dominance for negative expressions use two facial expressions (Prete et al., 2014b, 2015a; Burt and Hausmann, 2019; Bublatzky et al., 2020; Lai et al., 2020) than six (Stanković and Nešić, 2020). As such, other contributing factors must be considered.

Another factor is the particular task that subjects perform. Studies of hemisphere asymmetries in facial expression processing have employed a wide variety of tasks. Some studies ask subjects to decide which of two faces is more emotional (Bourne, 2010; Innes et al., 2016; Hausmann et al., 2021) or more like a particular emotion (Jansari et al., 2011; Rahman and Anchassi, 2012); others ask whether a face is emotional or not (Najt et al., 2013; Stanković and Nešić, 2020), a positive or negative expression (Lichtenstein-Vidne et al., 2017; Kajal et al., 2020), or the same or different expression compared to another (Mattavelli et al., 2013, 2016; Flack et al., 2015; Sliwinska and Pitcher, 2018). Of note, none of these tasks require subjects to explicitly identify different facial expressions (i.e., respond whether an expression is happy, angry, and sad, etc.), and studies that do require subjects to explicitly identify facial expressions are rare. Indeed, of studies that include all six emotional expressions, Abbott et al. (2014) and Ouerchefani et al. (2021) are the only ones in which subjects are asked to identify facial expressions in photographs. Both studies compare right and left hemisphere damage patients to control subjects and observe equivalent impairment for right and left hemisphere damage patients relative to controls for positive and negative expressions. In contrast, studies that include all six expressions and use tasks that do not require explicit identification (e.g., Bourne, 2010; Najt et al., 2013) observe right hemisphere dominance for most negative expressions.

Explicit identification of facial expressions in the context of multiple expressions may involve left hemisphere regions to a greater extent than tasks that do not require identification, due to the influence of linguistic processes that function dominantly in the left hemisphere. The influence of linguistic categories on left hemisphere facial expression processing was demonstrated in a study by Burt and Hausmann (2019) in which healthy subjects were sensitive to the linguistic category (happy vs. sad, angry vs. fearful) of facial expressions when they were presented to the left hemisphere but not when they were presented to the right. The researchers conclude that category-based processing of facial expressions is due to left hemisphere involvement. As such, tasks that require identifying the linguistic category of facial expressions may be more likely to involve left hemisphere regions than tasks that do not require this information.

The present research investigated this possibility in two experiments. Both experiments included all six basic emotional facial expressions presented to the right or left hemisphere using a divided-visual field paradigm. In Experiment 1, participants identified emotional expressions by pushing a key corresponding to one of six labels. In Experiment 2, participants detected emotional expressions by pushing a key corresponding to whether an expression was emotional or not. Left hemisphere dominance in negative expression processing was expected during Experiment 1, when an identification task was used, but not during Experiment 2, when a detection task was used.

## Experiment 1

### Method

#### Participants

Participants were 65 young adults (mean age = 19.39 years, *SD* = 1.09) recruited from Introduction to Psychology courses at Macalester College and the larger Macalester student body. All participants were right-handed as determined by the Edinburgh Inventory (Oldfield, 1971; mean laterality quotient = 0.804, *SD* = 0.166) and had normal or corrected-to-normal vision. Half (*N* = 33) identified as female and half (*N* = 32) identified as male. The majority (*N* = 39) identified as White, 13 identified as East Asian, 8 identified as Hispanic/Latinx, 4 identified as Black, and 1 identified as Middle Eastern. Participants were compensated with cash or extra credit in a course.

#### Design

The experiment employed a 6 × 2 design including expression (angry vs. disgusted vs. fearful vs. happy vs. sad vs. surprised) and hemisphere (left vs. right) as within-subjects independent variables. The dependent variable was percent correct.

#### Materials

Stimuli were black and white photographs of faces displaying six different expressions (angry, disgusted, fearful, happy, sad, and surprised) taken from the Pictures of Facial Affect database (Ekman and Freisen, 1976). Twelve faces (six female; six male) were included for each expression for a total of 72 emotional facial expressions. Faces were presented on a black background, subtended a visual angle of approximately 5° × 4° in vertical and horizontal dimensions, respectively, and were presented in the left or right visual field such that the center of each was approximately 6° from the center of the display and the inner edge never appeared closer than 4° from the center. Participants placed their chins in a chinrest to keep their eyes approximately 57 cm from the computer screen. Response recording and stimulus presentation were controlled using PsyScope (Cohen et al., 1993) on a Macintosh computer.

#### Procedure

Participants were tested individually in single sessions that lasted approximately 30 min. The expression identification task began immediately after participants were informed about the nature of the study and provided their consent to participate. Each trial began with the presentation of a fixation cross (+) in the center of the screen for 2,000 ms and was followed by the presentation of a face in the left or right visual field for 183 ms. After the face presentation, the center fixation cross remained on the screen until the participant pushed a key indicating their response and ended the trial. Participants responded using the “q,” “w,” “e,” “I,” “o,” and “p” keys to indicate angry, disgusted, sad, fearful, surprised, and happy expressions, respectively. Expression labels were provided above each key, and participants rested three fingers of their left (“q,” “w,” and “e”) and right (“I,” “o,” and “p”) hands on the keys throughout the task. Participants were instructed to keep their eyes focused on the fixation cross throughout, and to respond as quickly and accurately as possible when each face appeared. Ten practice trials were administered before the experimental trials began to ensure that participants understood the task instructions and familiarize them with the brief divided-visual field presentations and response keys. Experimental trials were administered in two blocks of 72 trials each, 12 for each of the six facial expressions, with half presented in the left visual field and half presented in the right. Stimuli were the same in each block but were presented in different orders that were randomized such that no more than three of the same type of expression or visual field were presented in a row.

### Results

**FIGURE 1:**
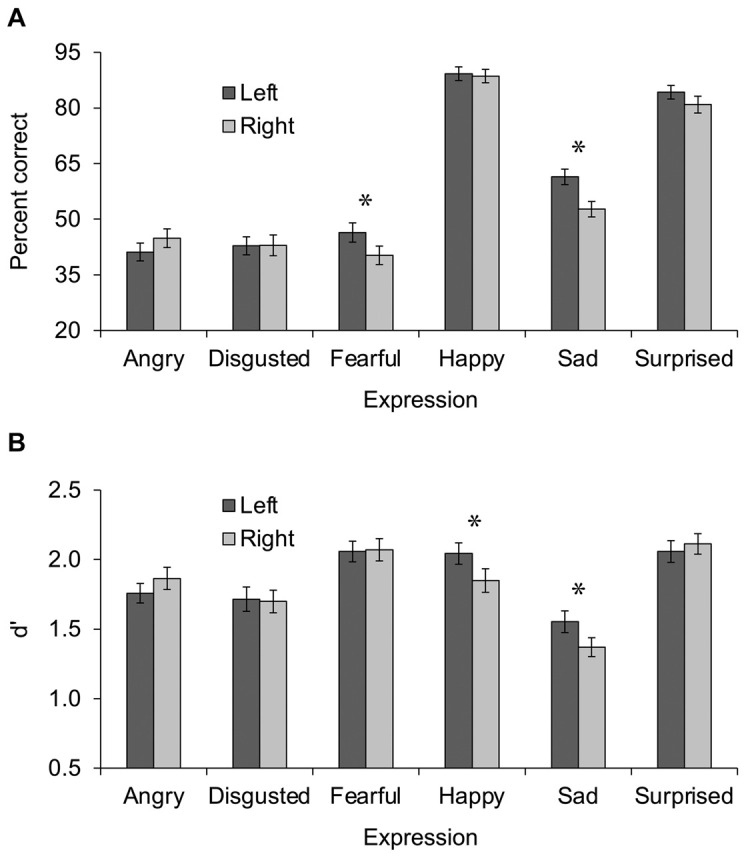
Performance during Experiment 1 **(A)** and Experiment 2 **(B)** presented as a function of expression (angry vs. disgusted vs. fearful vs. happy vs. sad vs. surprised) and hemisphere (left vs. right). Error bars display standard error of the mean. Asterisks indicate significant differences between left and right hemispheres.

Percent correct was analyzed in a 6 × 2 repeated-measures analysis of variance (ANOVA) with expression (angry vs. disgusted vs. fearful vs. happy vs. sad vs. surprised) and hemisphere (left vs. right) as independent variables. Results are shown in Figure 1A. A main effect of expression was observed, *F*(5, 60) = 147.84, *p* < 0.001, η_p_^2^ = 0.925, 95% CI (0.879, 0.941), in which happy expressions were identified most accurately (*M* = 89%, *SD* = 13%), followed by surprised (*M* = 83%, *SD* = 15%), then sad (*M* = 57%, *SD* = 14%), then angry (*M* = 43%, *SD* = 17%), disgusted (*M* = 43%, *SD* = 19%), and fearful (*M* = 43%, *SD* = 19%). A main effect of hemisphere in which left hemisphere trials (*M* = 61%, *SD* = 10%) were more accurate than right hemisphere trials (*M* = 58%, *SD* = 11%) was also observed, *F*(1, 64) = 7.71, *p* = 0.007, η_p_^2^ = 0.108, 95% CI (0.008, 0.258). Most important, the main effects were qualified by a significant interaction of expression x hemisphere, *F*(5, 60) = 3.91, *p* = 0.004, η_p_^2^ = 0.246, 95% CI (0.033, 0.364). Uncorrected *post hoc t* tests comparing the left and right hemisphere in each expression revealed greater percent correct in the left than right hemisphere for fearful, *t*(64) = 3.00, *p* = 0.004, *d* = 0.365, 95% CI (0.090, 0.493), and sad, *t*(64) = 3.85, *p* < 0.001, *d* = 0.433, 95% CI (0.193, 0.745), expressions, and no difference between hemispheres for the other expressions, all *p*s > 0.065.

Response times were analyzed in the same way as percent correct, after excluding incorrect responses and outliers below 250 ms or above 2.5 standard deviations of the participant’s mean, and replacing empty cells [8/780 cells (1%)] with the grand mean. Although this analysis is compromised by the unequal number of items contributing to different cell values, it is useful for assessing the extent to which the percent correct results may have been affected by a speed-accuracy tradeoff. Importantly, results were in line with results from the analysis of percent correct, indicating that no speed-accuracy tradeoff was present. Similar to the analysis of percent correct, a main effect of expression was observed, *F*(5, 60) = 112.15, *p* < 0.001, η_p_^2^ = 0.903, 95% CI (0.844, 0.924), in which happy expressions were identified fastest (*M* = 1,072 ms, *SD* = 215 ms) and followed by surprised (*M* = 1,477 ms, *SD* = 322 ms), then angry (*M* = 1,601 ms, *SD* = 396 ms), sad (*M* = 1,746 ms, *SD* = 379 ms), disgusted (*M* = 1,824 ms, *SD* = 434 ms), and fearful (*M* = 1,861 ms, *SD* = 413 ms). Also similar to the analysis of percent correct, a main effect of hemisphere was observed in which left hemisphere trials (*M* = 1,562 ms, *SD* = 271 ms) were faster than right hemisphere trials (*M* = 1,631 ms, *SD* = 280 ms), *F*(1, 64) = 11.41, *p* = 0.001, η_p_^2^ = 0.151, 95% CI (0.026, 0.307). Finally, although the interaction of expression x hemisphere was not significant, *F*(5, 60) = 1.09, *p* = 0.376, η_p_^2^ = 0.083, 95% CI (0, 0.167), the direction of mean differences was in line with the percent correct results, most importantly for fearful and sad facial expressions (see Table 1).

**TABLE 1:** Mean response times during Experiments 1 and 2.

Expression	Experiment 1				Experiment 2			
	Hemisphere				Hemisphere			
	Left		Right		Left		Right	
Angry	1,573	(491)	1,629	(448)	872	(204)	850	(207)
Disgusted	1,767	(477)	1,881	(511)	854	(164)	873	(198)
Fearful	1,814	(462)	1,908	(524)	817	(161)	804	(155)
Happy	1,062	(247)	1,081	(230)	819	(133)	832	(156)
Sad	1,719	(407)	1,772	(441)	903	(214)	911	(198)
Surprised	1,438	(332)	1,517	(379)	823	(163)	811	(178)

*Response times are provided in milliseconds. Parentheses indicate standard deviation of the mean.*

### Discussion

Experiment 1 examined hemisphere asymmetries during a facial expression identification task in which participants identified the six basic facial expressions. In line with the prediction that, due to its linguistic nature, an identification task should produce left hemisphere dominance for negative expressions, fearful, and sad expressions exhibited greater percent correct during left than right hemisphere presentations. Critically, although not all effects were significant in the analysis of response times, mean differences were in the same direction as those for percent correct, indicating that no speed-accuracy tradeoff was present.

The finding of a left-hemisphere advantage for fearful and sad expressions contrasts with predictions made by the RHH, VSH, and AWH, all of which predict a right hemisphere advantage for these negative expressions. Moreover, the lack of hemisphere asymmetry for any of the other expressions also contrasts with previous theories, albeit for different reasons: the RHH predicts a right hemisphere advantage for all emotional expressions; the VSH predicts a left hemisphere advantage for positive expressions such as happy and surprised; the AWH predicts a left hemisphere advantage for angry expressions. None of these predictions were supported.

Experiment 2 examined whether the left hemisphere advantage for negative fearful and sad expressions observed in Experiment 1 was due to the use of an identification task by testing an emotion detection task instead. Detecting whether a facial expression is emotional or not does not require identifying a linguistic category for the emotion, although this likely happens at least some of the time. Therefore, if the left hemisphere dominance observed in Experiment 1 was due to performance of an identification task, it should not be observed when emotion detection is performed.

In addition, it is important to note that response hand and key meaning were not counterbalanced in Experiment 1. That is, all participants always used their left hand for angry, disgusted, and sad responses and their right hand for fearful, surprised, and happy responses. Although it seems unlikely that response hand affected the pattern of results, given that sad and fearful expressions exhibited a similar hemisphere asymmetry using different response hands, it is nonetheless important to directly examine this possibility. Thus, Experiment 2 counterbalanced response hand and key meaning across and within participants and tested the effects.

## Experiment 2

### Method

#### Participants

Participants were 64 young adults (mean age = 19.82 years, *SD* = 1.33) recruited from Introduction to Psychology courses at Macalester College and the larger Macalester student body. None took part in Experiment 1. All were right-handed as determined by the Edinburgh Inventory (Oldfield, 1971; mean laterality quotient = 0.792, *SD* = 0.162) and had normal or corrected-to-normal vision. Half (*N* = 33) identified as female and half (*N* = 31) identified as male. The majority (*N* = 39) identified as White, 12 identified as East Asian, 7 identified as mixed race, 3 identified as Hispanic/Latinx, 2 identified as Southeast Asian, and 1 identified as Black. Participants were compensated with cash or extra credit in a course.

#### Design

The experiment employed a 6 × 2 design including expression (angry vs. disgusted vs. fearful vs. happy vs. sad vs. surprised) and hemisphere (left vs. right) as within-subjects independent variables. The dependent variable was sensitivity measured by d’ (Stanislaw and Todorov, 1999).

#### Materials and Procedure

The materials and procedure were identical to those used in Experiment 1 except for two important differences. For one, instead of identifying facial expressions, participants decided whether expressions were emotional or not. Participants responded with the “o” and “p” keys using their left hand during one block and their right hand during the other block. The mapping of response hand to block, and the meaning of the keys (emotional or not emotional), were counterbalanced across participants. In addition, in order to make the detection task possible, 72 non-emotional (neutral) facial-expression trials (36 in each visual field) were included with the 72 emotional facial-expression trials from Experiment 1 for a total of 144 trials per block. The 72 non-emotional trials were comprised of 12 neutral facial expressions repeated six times per block. As in Experiment 1, stimuli were the same in each block but were presented in different orders that were randomized such that no more than three of the same type of expression or visual field were presented in a row.

### Results

d’ was calculated according to Stanislaw and Todorov (1999) using “emotional” responses to emotional facial expressions as hits and “emotional” responses to non-emotional facial expressions as false alarms. Because estimates of d’ are undefined when hit or false alarm proportions are 1 or 0, values of 0 were converted to 1/(2*N*) and values of 1 were converted to 1 – 1/(2*N*), with *N* referring to the number of trials contributing to the proportion, as recommended by Macmillan and Creelman (1991).

d’ scores were analyzed in a 6 × 2 repeated-measures ANOVA with expression (angry vs. disgusted vs. fearful vs. happy vs. sad vs. surprised) and hemisphere (left vs. right) as independent variables. Results are shown in Figure 1B. A main effect of expression was observed, *F*(5, 59) = 28.85, *p* < 0.001, η_p_^2^ = 0.710, 95% CI (0.547, 0.770), in which d’ scores were highest for fearful (*M* = 2.06, *SD* = 0.570) and surprised (*M* = 2.09, *SD* = 0.548) expressions, followed by happy (*M* = 1.95, *SD* = 0.589), then angry (*M* = 1.81, *SD* = 0.521), then disgusted (*M* = 1.71, *SD* = 0.594), then sad (*M* = 1.46, *SD* = 0.504). The main effect of hemisphere was not significant, *F*(1, 63) < 1, η_p_^2^ = 0.010, 95% CI (0, 0.105), however, the interaction of expression x hemisphere was, *F*(5, 59) = 4.36, *p* = 0.002, η_p_^2^ = 0.270, 95% CI (0.048, 0.389). Uncorrected *post hoc t* tests comparing the left and right hemisphere in each expression revealed greater d’ scores in the left than right hemisphere for happy, *t*(63) = 2.90, *p* = 0.005, *d* = 0.364, 95% CI (0.083, 0.502), and sad, *t*(63) = 2.47, *p* = 0.016, *d* = 0.303, 95% CI (0.052, 0.561), expressions, and no difference between hemispheres for the other expressions, all *p*s > 0.159.

In order to assess any potential effects of response hand and key meaning, d’ scores were secondarily analyzed in a 6 (expression) × 2 (hemisphere) × 2 × 2 × 2 repeated-measures ANOVA that included response hand (left vs. right) as a within-subjects independent variable, and hand-to-block mapping (left hand-block 1, right hand-block 2 vs. right hand-block 1, left hand-block 2) and key meaning (emotional = “p,” not emotional = “0” vs. emotional = “o,” not emotional = “p”) as between-subjects independent variables. None of the interactions including response hand, response hand × hand-to-block mapping, or key meaning were significant, all *p*s > 0.133.

As in Experiment 1, an analysis of response times was conducted in order to assess any speed-accuracy tradeoff. Response times for correct emotional facial expressions (hits), excluding outliers below 250 ms or above 2.5 standard deviations of the participant’s mean, were analyzed in a 6 × 2 repeated-measures ANOVA with expression (angry vs. disgusted vs. fearful vs. happy vs. sad vs. surprised) and hemisphere (left vs. right) as independent variables. Importantly, results were in line with results from the analysis of d’, indicating that no speed-accuracy tradeoff was at play. Similar to the analysis of d’, a main effect of expression was observed, *F*(5, 59) = 14.00, *p* < 0.001, η_p_^2^ = 0.543, 95% CI (0.322, 0.634), in which response times were fastest for fearful (*M* = 810 ms, *SD* = 149 ms) and surprised (*M* = 817 ms, *SD* = 159 ms) expressions, followed by happy (*M* = 825 ms, *SD* = 136 ms), then angry (*M* = 861 ms, *SD* = 194 ms) and disgusted (*M* = 863 ms, *SD* = 167 ms), then sad (*M* = 907 ms, *SD* = 192 ms). Neither the main effect of hemisphere, *F*(1, 63) < 1, η_p_^2^ = 0.000, 95% CI (0, 0.052), nor the interaction of expression × hemisphere, *F*(5, 59) < 1, η_p_^2^ = 0.075, 95% CI (0, 0.155), were significant, however, the direction of the means was in line with the d’ results, most importantly for happy and sad facial expressions (see Table 1).

Potential effects of response hand and key meaning on response times were assessed in the same way as for d’, in a 6 (expression) × 2 (hemisphere) × 2 × 2 × 2 repeated-measures ANOVA that included response hand (left vs. right) as a within-subjects independent variable, and hand-to-block mapping (left hand-block 1, right hand-block 2 vs. right hand-block 1, left hand-block 2) and key meaning (emotional = “p,” not emotional = “0” vs. emotional = “o”, not emotional = “p”) as between-subjects independent variables. None of the interactions including response hand, response hand × hand-to-block mapping, or key meaning were significant, all *p*s > 0.070.

In order to compare the results from Experiments 1 and 2, percent correct scores from Experiment 1 and d’ scores from Experiment 2 were converted to *z* scores and analyzed in a 6 × 2 × 2 repeated-measures ANOVA with expression (angry vs. disgusted vs. fearful vs. happy vs. sad vs. surprised) and hemisphere (left vs. right) as within-subjects independent variables, and task (identification vs. detection) as a between-subjects variable. The interaction of expression × hemisphere × task was significant, *F*(5, 123) = 2.68, *p* = 0.025, η_p_^2^ = 0.098, 95% CI (0, 0.174). This interaction was explored with *post hoc* repeated-measures ANOVAs (uncorrected) including hemisphere and task for each expression. Fearful and happy facial expressions exhibited effects that varied by task. Performance on fearful facial expressions was greater in the left than right hemisphere during the identification task but not during the detection task, *F*(1, 127) = 4.36, *p* = 0.037, η_p_^2^ = 0.034, 95% CI (0, 0.113), for the hemisphere × task interaction. Performance on happy facial expressions was greater in the left than right hemisphere during the detection task but not during the identification task, *F*(1, 127) = 5.28, *p* = 0.023, η_p_^2^ = 0.040, 95% CI (0, 0.124), for the hemisphere × task interaction. Angry and sad facial expressions exhibited effects of hemisphere that did not vary by task. Performance on angry expressions was greater in the right (*M* = -0.265, *SD* = 0.916) than left hemisphere (*M* = –0.416, *SD* = 0.838) in a main effect of hemisphere, *F*(1, 127) = 4.22, *p* = 0.042, η_p_^2^ = 0.032, 95% CI (0, 0.112), with no interaction of hemisphere × task, *F*(1, 127) < 1, η_p_^2^ = 0.000, 95% CI (0, 0.024). Performance on sad expressions was greater in the left (*M* = –0.187, *SD* = 0.841) than right hemisphere (*M* = –0.490, *SD* = 0.766) in a main effect of hemisphere, *F*(1, 127) = 18.54, *p* < 0.001, η_p_^2^ = 0.127, 95% CI (0.038, 0.237), with no interaction of hemisphere × task, *F*(1, 127) < 1, η_p_^2^ = 0.001, 95% CI (0, 0.037). Disgusted and surprised facial expressions did not exhibit effects of hemisphere or hemisphere × task, all *p*s > 0.082.

### Discussion

Experiment 2 examined hemisphere asymmetries during an emotion detection task in which participants decided whether facial expressions were emotional or not. It was predicted that this task would not produce the left hemisphere advantage for negative expressions of fear and sadness seen in Experiment 1. In line with this prediction, fearful facial expressions did not exhibit a left hemisphere advantage. In contrast to this prediction, however, d’ for sad facial expressions was greater in the left than right hemisphere, indicating more general left hemisphere involvement in the processing of sad expressions. Experiment 2 also revealed greater d’ in the left than right hemisphere for happy facial expressions, which is in line with predictions of the VSH. However, except for angry facial expressions, which only exhibited a hemisphere effect when Experiments 1 and 2 were combined, no expressions exhibited a right hemisphere advantage, which is in contrast to predictions made by the VSH, as well as the RHH and AWH. The right hemisphere advantage observed across experiments for angry expressions is in line with the RHH and VSH but contrasts with the AWH. Critically, although response times in Experiment 2 did not exhibit significant effects of hemisphere, mean differences were in the same direction as those for d’, indicating that no speed-accuracy tradeoff was present.

Experiment 2 improved on Experiment 1 by counterbalancing the response hand and key meaning within and across participants. Importantly, no effects of response hand or key meaning were observed in either d’ or response time measures, indicating that these variables did not have a significant influence on performance. Of course, it is still possible that they had an influence in Experiment 1, however, this possibility seems less likely given their lack of effect in Experiment 2.

## General Discussion

Three major theories of hemisphere asymmetries in emotion processing—the RHH, VSH, and AWH—predict right hemisphere dominance for negative facial expressions of disgust, fear, and sadness. This is observed in many studies (e.g., Bourne, 2010; Kumar and Srinivasan, 2011; Rahman and Anchassi, 2012; Innes et al., 2016; Damaskinou and Watling, 2018; Kajal et al., 2020; Hausmann et al., 2021), however, some studies observe left hemisphere dominance for one or more of these expressions (Prete et al., 2014b, 2015a; Burt and Hausmann, 2019; Bublatzky et al., 2020; Lai et al., 2020; Stanković and Nešić, 2020). The present research investigated whether tasks that require identifying the linguistic category of facial expressions are more likely to produce left hemisphere dominance than tasks that do not require this information. In line with this idea, fearful facial expressions exhibited a left hemisphere advantage during an identification task (Experiment 1) but not during an emotion detection task (Experiment 2). In contrast to this idea, however, sad expressions exhibited a left hemisphere advantage during both identification and detection tasks, and happy facial expressions exhibited a left hemisphere advantage during the detection task but not during the identification task. As such, the present study provides partial support for the idea that identification increases left hemisphere involvement and raises additional questions.

One question is why fearful facial expressions were the only expressions that exhibited greater left hemisphere involvement in the identification task than the detection task, when it was predicted that all negative expressions, as well as positive expressions, would show this effect. Research suggests that highly arousing emotions such as fear are processed rapidly by the amygdala and related subcortical regions (Le Doux, 2007; Lindquist et al., 2012). Indeed, Fusar-Poli et al. (2009) observe greater bilateral amygdala activity for fearful expressions compared to other expressions in a meta-analysis of fMRI studies of facial expression processing. It is possible, therefore, that the detection of fear relies on subcortical structures that do not exhibit a hemisphere asymmetry while the identification of fear requires more elaborate processing by cortical systems that do exhibit a hemisphere asymmetry. This is similar to a model proposed by Shobe (2014) in which emotional stimuli are processed initially by subcortical regions, and secondarily by cortical regions in the right and left hemispheres that process the emotional information more fully. Shobe argues that subcortical regions are right lateralized, however, which is not in line with the lack of hemisphere asymmetry for fearful expressions in the detection task. In contrast to fear, less arousing emotions such as happy and sad may rely on cortical systems for both identification and detection. Indeed, the similar hemisphere asymmetry observed in the identification and detection tasks for sad facial expressions is in line with this idea. Nonetheless, the effect of task observed for happy facial expressions (left hemisphere advantage during detection but not identification) is not. Thus, this cannot be the complete explanation.

Although a similar hemisphere asymmetry in identification and detection tasks for sad facial expressions is in line with the idea that low-arousal emotions rely on the same cortical systems regardless of task, the left hemisphere advantage in both tasks contrasts with the hypothesis that left hemisphere involvement for negative facial expressions is due to the need for linguistic categorization, as this is not needed for emotion detection. Results from Lai et al. (2020) suggest another possible explanation. In this study, N170 latencies in the left hemisphere were shorter for sad than happy expressions when faces were presented for a brief amount of time (14 ms), implying that left hemisphere regions play a role in the early processing of sad facial expressions. If so, this could explain the left hemisphere advantage for sad facial expressions in the detection task.

Nonetheless, the question of why left hemisphere regions might be important for processing sad facial expressions remains. Sadness is characterized by the AWH as a “withdrawal” emotion that should evoke right hemisphere dominance, and indeed, as noted previously, there is much research to support this interpretation (e.g., Bourne, 2010; Kumar and Srinivasan, 2011; Najt et al., 2013; Innes et al., 2016). However, others have argued that sad facial expressions may evoke prosocial or caregiving responses in the viewer (Tooby and Cosmides, 1990; Lazarus, 1991). From this perspective, sadness could be reconceived as an “approach” emotion that should, according to the AWH, produce left hemisphere dominance. In line with the idea that sad facial expressions elicit a prosocial/caregiving response, Colasante et al. (2017) observed a larger N170 response in adult participants for sad infant faces than for sad adult faces or happy faces. Critically, however, the N170 response was in the right hemisphere rather than the left, contradicting the idea that sadness is left dominant. Clearly further research will be necessary to explain this effect.

In contrast to left hemisphere dominance for sad facial expressions, left hemisphere dominance for happy facial expressions is predicted by both the VSH and AWH, as happy expressions are both positive and approach-oriented. The left hemisphere advantage for happy facial expressions observed during the detection task is in line with this prediction. It is unclear, however, why this effect was not observed during the identification task, which was hypothesized to require greater left hemisphere involvement than the detection task due to its linguistic nature. Future research will be required to fully understand hemisphere asymmetries in happy facial expression processing. Nevertheless, the present research highlights the complexity of the issue and the need to consider task demands.

In addition to considering task demands, future research should include all six basic emotional expressions, as was done in the present experiments. This is important because it increases left hemisphere involvement compared to only including two expressions (Abbott et al., 2014). Moreover, including all six basic emotional expressions in future research will increase the comparability of studies by ensuring that the same emotional expressions are tested. Currently, many studies only include two emotional facial expressions (Killgore and Yurgelun-Todd, 2007; Kumar and Srinivasan, 2011; Mattavelli et al., 2013, 2016; Prete et al., 2014a, 2015a, b, 2018; Colasante et al., 2017; Lichtenstein-Vidne et al., 2017; Damaskinou and Watling, 2018; Bublatzky et al., 2020; Kajal et al., 2020; Lai et al., 2020) and which two are included varies [e.g., sad (Damaskinou and Watling, 2018; Lai et al., 2020) vs. angry (Prete et al., 2014a, 2015a, b, 2018; Lichtenstein-Vidne et al., 2017)], making studies difficult to compare. In addition, studies including only two expressions rarely include expressions of disgust or surprise, and therefore, these expressions have not received as much attention as others. Neither disgust nor surprise exhibited a hemisphere asymmetry in the present experiments, but it will be crucial to know more about these expressions for a complete understanding of asymmetries in expression processing. Finally, future facial expression research should include all six expressions because tasks that require processing of six expressions are closer to real-life facial expression processing, which includes a wide variety of expressions, than tasks that only require two, and as such, provide a more realistic perspective on the systems and processes involved.

Future research will also benefit from the use of different methods for assessing hemisphere asymmetries in expression processing. A recent ERP study observed widespread bilateral activity during divided-visual-field presentations of facial expressions (Prete et al., 2018), calling into question the extent to which divided-field presentations preferentially activate left or right hemispheres. Moreover, this same study observed different support for the RHH and VSH depending on the measure examined, with behavioral performance supporting the VSH and ERP results supporting the RHH. As such, it cannot be assumed that neuroimaging methods will produce similar results as the present study. Other research indicates that whether an expression is perceived consciously or unconsciously affects the pattern of hemisphere asymmetries, and suggests that unconsciously perceived expressions may preferentially activate the left or right hemispheres more effectively than consciously perceived expressions (Prete et al., 2015a). Expressions in the present study were consciously perceived; thus future research may benefit from presentations in which the expression is perceived unconsciously.

In conclusion, the present research adds to a growing body of work on facial expression processing that does not align with major theories of hemisphere asymmetries in emotion processing (Killgore and Yurgelun-Todd, 2007; Tamietto et al., 2007; Rahman and Anchassi, 2012; Abbott et al., 2013; Najt et al., 2013; Prete et al., 2015a, b, 2018, 2019; Lai et al., 2020). Clear evidence for left hemisphere involvement in processing the negative facial expressions of fear and sadness was observed, which contrasts with predictions of previous theories and provides a foundation for further research into the nature of this role. The present research also provides clear evidence for the influence of task demands on hemisphere asymmetries for certain expressions, namely fearful and happy. Thus, task demands should be considered in future investigations.

## Data Availability Statement

The raw data supporting the conclusion of this article will be made available by the authors, without undue reservation.

## Ethics Statement

The studies involving human participants were reviewed and approved by the Psychology Review Board at Macalester College. The patients/participants provided their written informed consent to participate in this study.

## Author Contributions

EB was responsible for the conception and design of the experiments, the statistical analyses, and wrote the manuscript.

## Conflict of Interest

The author declares that the research was conducted in the absence of any commercial or financial relationships that could be construed as a potential conflict of interest.

## Publisher’s Note

All claims expressed in this article are solely those of the authors and do not necessarily represent those of their affiliated organizations, or those of the publisher, the editors and the reviewers. Any product that may be evaluated in this article, or claim that may be made by its manufacturer, is not guaranteed or endorsed by the publisher.
